# Association betweensubchondral bone sclerosis and pain in varus ankle osteoarthritis: a CT-based analysis using patient-reported outcomes

**DOI:** 10.1007/s00402-025-06096-0

**Published:** 2025-10-17

**Authors:** Dan Moriwaki, Tomoyuki Nakasa, Yasunari Ikuta, Saori Ishibashi, Satoru Sakurai, Taro Chujo, Nobuo Adachi

**Affiliations:** 1 Department of Orthopaedic Surgery, Hiroshima university, Hiroshima, Japan; 2 Department of Artificial Joints and Biomaterials, Hiroshima university, Hiroshima, Japan

**Keywords:** Ankle osteoarthritis, Pain, Subchondral bone sclerosis, Synovitis, Computed tomography

## Abstract

**Purpose:**

Although severe pain caused by progressive ankle osteoarthritis (OA) impairs patients’ quality of life, factors associated with severe pain in ankle OA are unknown. This study aimed to analyze the characteristics of pain and the association between pain, radiographic alignment, and computed tomography (CT) and magnetic resonance imaging (MRI) findings in patients with varus ankle OA.

**Methods:**

Seventy-five ankles from 73 patients with varus ankle OA who underwent surgery were retrospectively reviewed. Pain was evaluated using the self-administered foot evaluation questionnaire (SAFE-Q). Ankle alignment was assessed by radiography. Subchondral bone sclerosis was assessed by CT-derived Hounsfield unit (HU) ratio, and synovitis and bone marrow edema (BME) were assessed by MRI. The relationship between pain and imaging findings was analyzed.

**Results:**

Pain and pain-related scores in the SAFE-Q significantly correlated with the Takakura-Tanaka classification stage (*r* = − 0.529), osteophyte score (*r* = − 0.460), HU ratios (*r* = − 0.729), synovial thickness score (*r* = − 0.387), and BME area (*r* = − 0.475). Multivariate analysis revealed that high HU ratio, progressed OA stage, and thick synovium were significantly associated with severe pain. Notably, HU ratios showed moderate to strong correlations with pain, regardless of radiographic severity, even in the regions where joint space was radiographically preserved.

**Conclusions:**

Subchondral bone sclerosis, OA severity, and synovitis are significantly correlated with pain in patients with varus ankle OA. The novel finding is that HU ratios of subchondral bone correlate with pain irrespective of radiographic stage, suggesting that severe pain reflects hidden mechanical stress and cartilage degeneration associated with subchondral sclerosis. Varus ankle OA with severe pain should be managed early and appropriately, regardless of radiographic severity.

**Level of evidence:**

III.

## Introduction

Ankle osteoarthritis (OA) is a progressive degenerative disease of articular cartilage of the ankle joint, mainly caused by recurrent ankle sprain and fracture [1]. Varus deformity is common in ankle OA, bone abnormalities, chronic lateral ligament insufficiency, muscular imbalance, hindfoot varus alignment, or a combination of these factors can all contribute to a varus deformity [2]. Although its prevalence is approximately 1% in the adult population and is less common than hip or knee OA [3], ankle OA causes progressive joint deterioration with severe pain and functional disability, impairing quality of life to a degree comparable with severe hip OA, advanced renal failure, or congestive heart failure [4, 5]. Therefore, understanding the pathogenesis and characteristics of pain associated with ankle OA and performing appropriate pain management are crucial in developing effective treatment strategies. However, pain associated with OA is a complex outcome influenced by various factors, including cartilage degeneration, subchondral bone change, osteophyte formation, synovial inflammation, bone marrow edema (BME), ligamentous laxity, and sensory innervation [6–9]. Notably, factors that contribute strongly to ankle OA pain are unclear.

Patient-reported outcome measures (PROMs) are used globally in routine clinical practice and research to assess patients’ perceptions of disability, health, and quality of life [10]. The Self-Administered Foot Evaluation Questionnaire (SAFE-Q) is a PROM developed to assess health-related quality of life in individuals with foot- and ankle-related morbid conditions [11–13]. The main body of the SAFE-Q comprises 34 items providing five subscale scores: pain and pain-related, physical functioning and daily living, social functioning, shoe-related, and general health and well-being. The pain and pain-related subscale includes the visual analog scale (VAS) and various pain patterns. Previous reports reported an association between the degree of pain and intra-articular pathology using the SAFE-Q in patients with chronic lateral ankle instability (CLAI) and osteochondral lesion of the talus (OLT) [9, 14].

We hypothesized that the patient-reported pain via SAFE-Q could characterize ankle OA pain and that exploring the relationship between patient-reported pain and image findings could help identify key factors associated with OA pain. The primary aim of this study was to clarify the association between SAFE-Q pain scores and imaging-based parameters, including radiographic alignment, subchondral bone sclerosis on computed tomography (CT), and synovitis and BME on magnetic resonance imaging (MRI) in patients with varus ankle OA.

## Materials and methods

### Patient selection

Overall, 75 ankles of 73 patients treated surgically for ankle OA or chronic lateral ankle instability (CLAI) between April 2018 and April 2025 were retrospectively reviewed. The inclusion criteria were patients who had shown varus ankle OA on weight-bearing plain radiography, underwent both CT and MRI of the ankle, and completed the SAFE-Q preoperatively. Therefore, both ankle OA and CLAI accompanied by radiographic findings of varus OA were included, because CLAI is a frequent etiological factor in the development of varus ankle OA. To unify the pathology background of ankle OA as much as possible, the exclusion criteria were valgus ankle OA, concomitant osteochondral lesion of the talus (OLT) identified on CT or MRI, history of ankle joint fracture, revision surgery, talar necrosis, and systemic diseases such as rheumatoid arthritis. The patients comprised 32 men and 41 women, with a mean age of 57.3 ± 15.1 (range, 20–88) years. Two patients exhibited bilateral involvement. Of the 75 ankles included, 62 (82.7%) were associated with CLAI, and 13 (17.3%) were classified as primary ankle OA. This study was approved by the local ethics committee of our university (E-879), and written informed consent was obtained from all participants.

### Patient-reported outcome

The SAFE-Q questionnaire was administered to patients preoperatively, and patients completed the questionnaires themselves. The pain and pain-related subscales consisted of 9 questions. All questions, except question 3, were scored from 0 (severe pain) to 4 (no pain) points, with lower scores indicating worse disease status. For question 3, patients marked on the line, with 0 indicating “no pain” and 10 indicating the “worst pain imaginable”. The score for question 3 was calculated using the VAS value on a full scale of 10 cm and the formula: (10- VAS value) × 0.4. Pain and pain-related score was defined as (sum of points) × 25/(number of questions). Thus, the lowest possible score for each subscale was 0, and the highest was 100 points [11–13]. The median value of the pain and pain-related score was calculated; the group with a score lower than the median was defined as the severe pain group, and the group with a score higher than the median as the mild pain group. Other SAFE-Q subscales (physical function, social function, shoe-related, and general health) were also calculated but were not the main focus of this analysis.

### Physical and radiographic evaluation

The range of motion (ROM) of the ankles was physically measured using a goniometer before surgery. ROM measurements were performed by two senior orthopaedic surgeons with the patient in a supine position, with the knee fully extended for assessment of dorsiflexion and plantarflexion.

Radiographic parameters were measured preoperatively using plain weight-bearing anteroposterior and lateral radiographs of the ankle. The severity of ankle OA was graded using the Takakura-Tanaka classification: stage 1, no narrowing of the joint space, but early sclerosis and formation of osteophytes; stage 2, narrowing of the medial joint space; stage 3a, obliteration of the joint space was limited to the medial malleolus; stage 3b, the obliteration extended to the roof of the dome of the talus; and stage 4, obliteration of the whole joint space with complete bone contact [15, 16]. The tibial anterior surface angle, talar tilt angle (TTA), tibiomedial malleolar angle (TMMA), and tibial lateral surface angle were measured according to a previously reported method [17]. The severity of the osteophyte was graded using the Kraus classification, which categorizes osteophytes in 10 regions of the ankle joint (medial, lateral, anterior, and posterior of the tibia and talus; distal fibula; and posterior talus at the subtalar joint) on a 0–3 scale (0, absent; 1, small; 2, moderate; 3, large) [18]. The osteophyte score was defined as the sum of the severity grades in all 10 regions, with a possible range of 0–30.

### CT evaluation

CT was performed for preoperative evaluation. Images of the ankles in the coronal, sagittal, and axial planes were acquired using a 64-multi-detector-row CT scanner (Light-Speed QX/I; GE Healthcare, Chicago, IL, USA). After scanning, two-dimensional images were reconstructed with a 25-cm field of view, 1.25-mm retrospective slice thickness, and 0.63-mm overlap. The Hounsfield unit (HU) values were quantitatively evaluated on the tibial, talar, and fibular articular surfaces of the tibiotalar joint, medial gutter, and lateral gutter. The HU is defined based on a linear transformation of the original linear attenuation coefficient measurements to a scale where the radiant density of distilled water at standard temperature and pressure is 0 and that of air is −1000 [19]. HU values of the subchondral bone highlight the chronic mechanical stress on the joint and detect small changes in the osteochondral unit of the joint [20, 21].

**Fig. 1:**
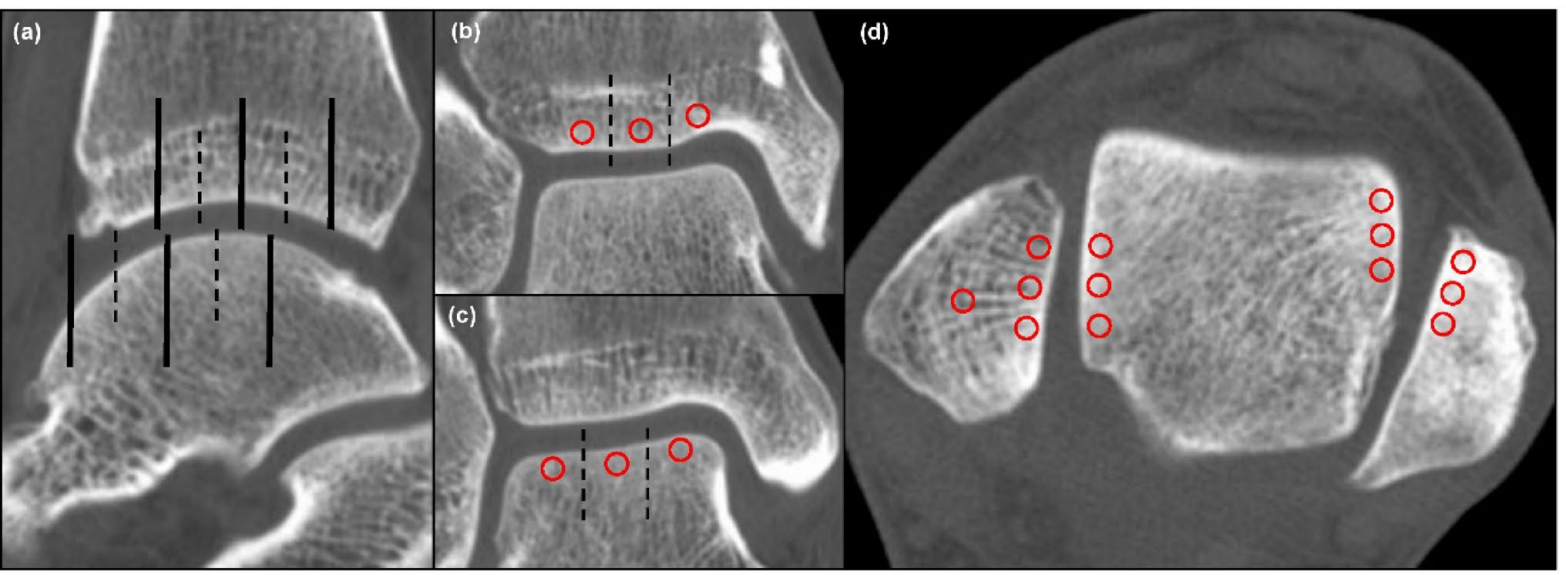
Measurement of the Hounsfield Unit values. **a** Scan lines in the sagittal slice of computed tomography. Dashed lines indicate the borders of the anterior, middle, and posterior parts. Black lines indicate scan lines of the tibia and talus, respectively. Three regions of interest (ROIs) (red circles) were set at the subchondral bone in the tibial plafond (**b**) and talar dome (**c**) in each coronal slice. Dashed lines indicate the borders of the medial, central, and lateral parts. **d** ROIs (red circles) of the medial and lateral gutters, and the center of the fibula as a reference

**Fig. 2:**
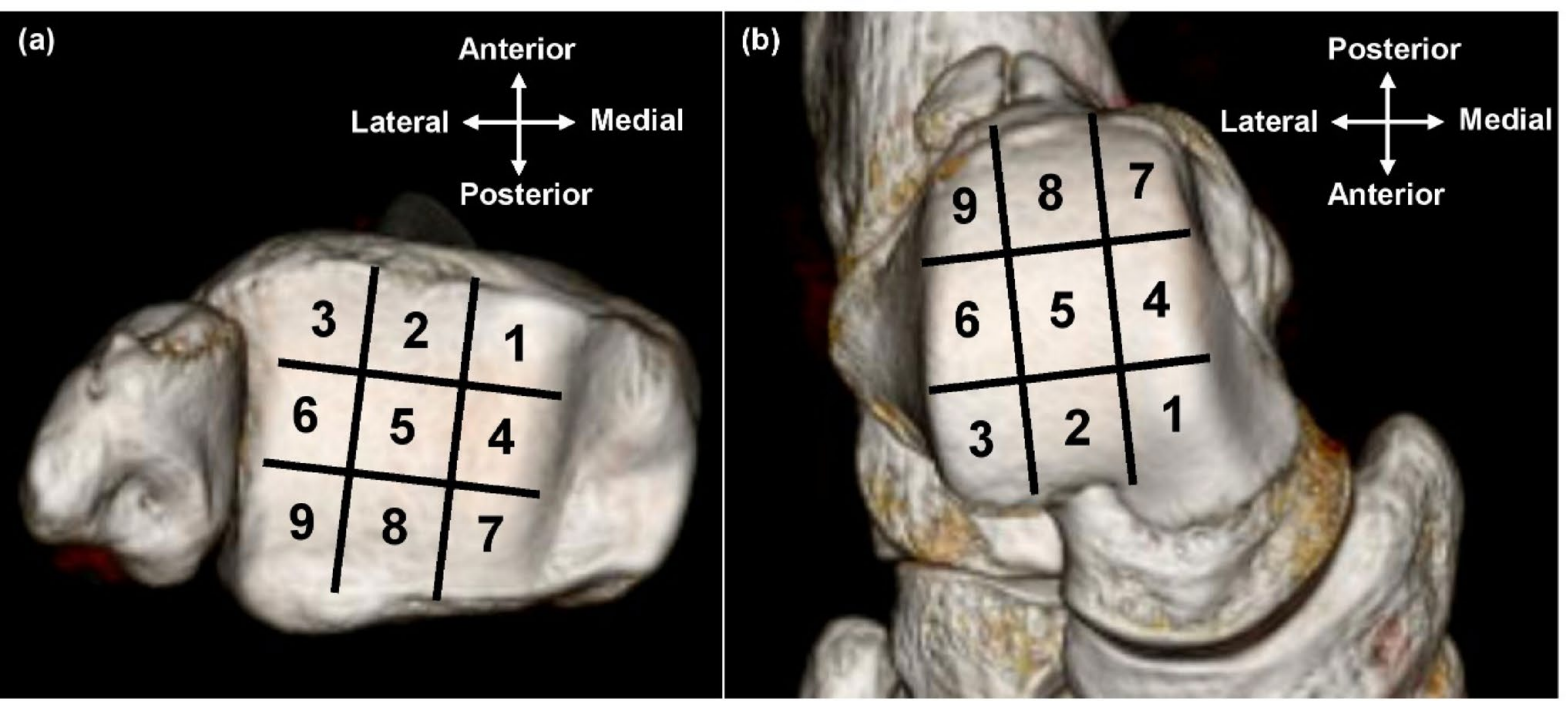
Nine areas in the tibial plafond (**a**) and talar dome (**b**). The Hounsfield Unit values were measured in each of the nine areas

Sagittal slices through the center of the tibial plafond were obtained from CT data. The articular surfaces of the tibia and talus were divided into three parts: anterior, middle, and posterior. A scan line was established through the midpoint of each part, and six coronal slices were obtained (Fig. 1a). Each coronal slice was divided into three parts: medial, central, and lateral. One region of interest (ROI) was manually set just below the subchondral bone plate in each part, yielding three ROIs per coronal slice, and mean HU values in the ROIs were measured (Fig. 1bc). When bone cysts were present, ROIs were placed to avoid cystic areas. Consequently, the HU values of the tibiotalar joint were measured at nine locations (Zone 1- 9) in the tibial plafond and talar dome, respectively (Fig. 2). HU values of the medial gutter were measured according to a previous report [21], at the axial level corresponding to the anterior part of the oval surface for insertion of the talotibial component of the deltoid ligament at the talus. HU values of the lateral gutter were measured as in the medial gutter; the three points of the ROI were set in the subchondral bone of the joint surfaces of the talus, and the three other points were set at the opposite side of the fibula by axial sectioning (Fig. 1d). All ROIs were in a 5.5-mm2 circle. The HU values at the center of the fibula were measured in the same slice of the medial gutter as a reference [21]. The HU ratio was calculated by dividing the HU value of each region by that of the center of the fibula. The mean HU ratios in zones 1, 4, and 7, zones 2,5,8, and zones 3,6,9 were defined as the HU ratios in the medial, central, and lateral parts, respectively. Three-dimensional reconstructions were performed through a volume-rendering method using commercially available software (SYNAPSE VINCENT, FUJIFILM, Tokyo, Japan).

### MRI evaluation

MRI was performed using a Signa 1.5-Tesla device or a Signa HDxt 3.0-T device (GE Yokogawa Medical Systems Ltd) with a wraparound surface coil designed for the ankle joint. Axial, coronal, and sagittal fat-suppressed turbo spin-echo T2-weighted images were used to evaluate synovitis and BME. The severity of synovitis was assessed according to a previous report [22]. In the axial plane, the ankle joint was divided into four compartments: the anterior recess, anteromedial gutter, anterolateral gutter, and posterior recess. Synovial thickness was semi-quantitatively classified with a grade of 0-2 points for each compartment. The synovial thickness score was calculated as the total severity of synovitis in the four compartments (range, 0- 8). BME was defined as the presence of a region of high signal intensity within the host marrow on T2-weighted images. On both the coronal and sagittal planes, the largest area of BME in the tibia, talus, and fibula was measured, using Image J Software (National Institutes of Health, Bethesda, MD, USA) according to previous reports [14, 23]. The mean BME areas in the coronal and sagittal planes of each bone were summed to assess the BME of the entire ankle joint (total BME).

### Statistical analysis

Between-group differences were calculated using the Mann-Whitney U test and Fisher’s exact test for categorical variables. Spearman’s correlation coefficient was used to explore the relationship between the two groups. Statistical significance was set at *P*< 0.05. Multiple regression analysis was performed to identify factors that significantly influence pain and pain-related scores. The independent variables included age, body mass index, TTA, Takakura-Tanaka classification stage, osteophyte score, mean HU ratio of all regions, synovial thickness score, and total BME. The multicollinearity diagnostic was performed using the variance inflation factor (VIF) for all independent variables. In the Spearman correlation and multiple regression analysis, ankles in stages 3 A and 3B were unified as stage 3 for the Takakura-Tanaka classification.

All measurements were performed by two independent observers and repeated twice by one observer. Intra- and inter-observer reliability was evaluated using the intra-class correlation coefficient (ICC). ICC values ≤0.5, between 0.5 and 0.75, between 0.75 and 0.9, and ≥0.90 indicated poor, moderate, good, and excellent reliability, respectively [24]. All statistical analyses were performed using EZR (Saitama Medical Center, Jichi Medical University, Saitama, Japan), which is a graphical user interface for R (The R Foundation for Statistical Computing, Vienna, Austria).

## Results

**Fig. 3:**
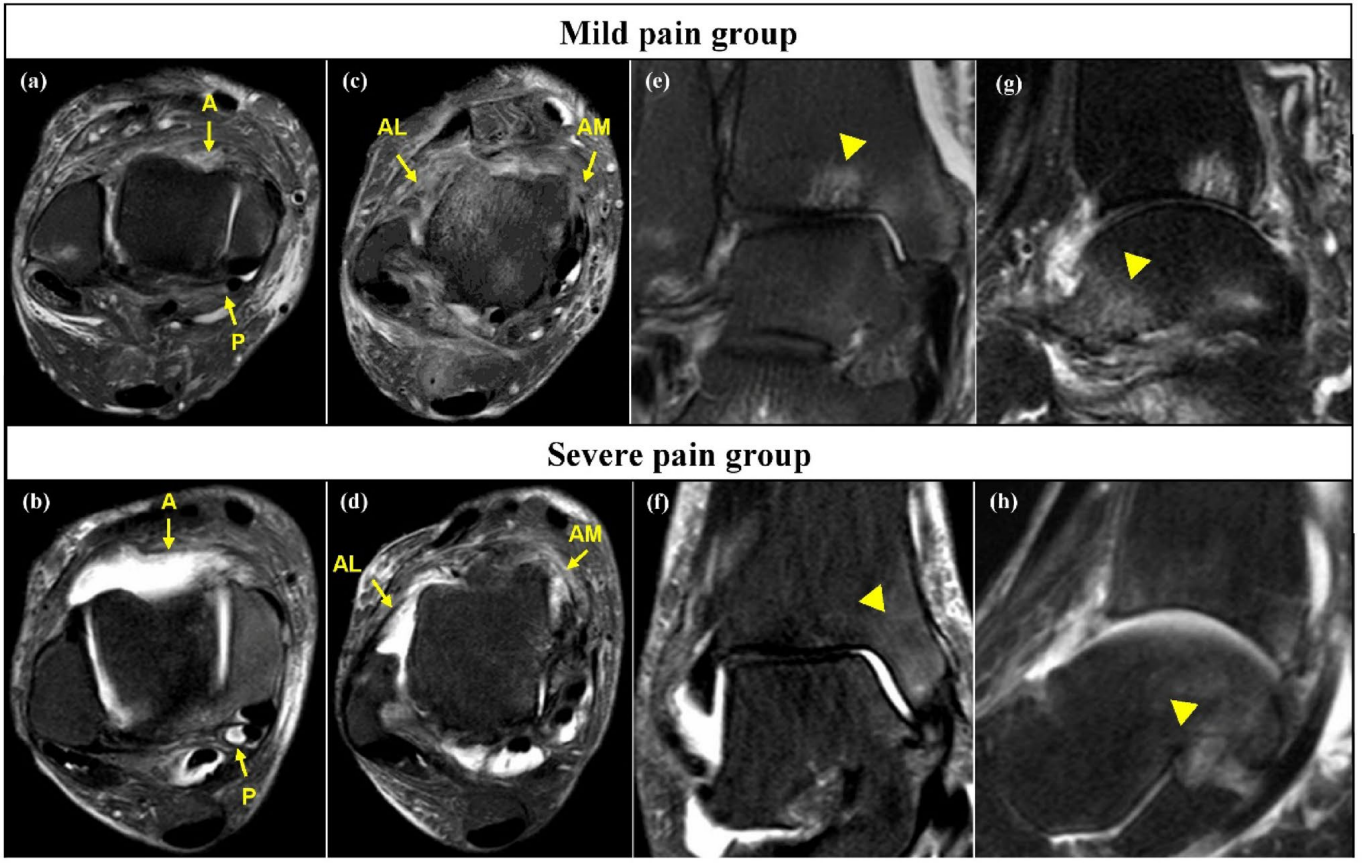
Representative magnetic resonance imaging of the mild pain and severe pain groups. **a**, **b** Axial slice showing the anterior (A) and posterior recess (P). **c**, **d** Axial slice showing anteromedial (AM) and anterolateral gutter (AL). **e**, **f** Coronal slice showing the largest bone marrow edema (BME) in the tibia. (g, h) Sagittal slice showing the largest BME in the talus. Arrowheads indicate BME

Table 1 summarizes the demographic characteristics, physical findings, and SAFE-Q subscale scores of the study cohort. Patients in the severe pain group were significantly older and had a lower BMI, lower SAFE-Q scores in all domains, and higher VAS compared with the mild pain group (all *P* < 0.05). Table 2 presents the radiographic and imaging findings, demonstrating that the severe pain group had more advanced OA stage, greater TTA and TMMA, larger osteophytes, higher HU ratios except for the fibula, thicker synovium (Fig. 3), and larger BME areas (all *P* < 0.05).

The correlations between pain and pain-related score on the SAFE-Q and each parameter are shown in Table 3. All HU ratios, except for the fibula, showed moderate to strong negative correlations with pain and pain-related scores. The osteophyte score and total BME showed moderate negative correlations, whereas age, TTA, TMMA, and synovial thickness scores showed weak negative correlations with pain and pain-related score. The results of the multiple regression analysis are shown in Table 4. All calculated VIF values, as described in the Materials and Methods section, were well below the commonly accepted threshold of 5, indicating no multicollinearity. A greater HU ratio, synovial thickness score, and Takakura-Tanaka classification grade were significant independent variables for lower pain and pain-related score.

Correlations between answers to questions on pain in the SAFE-Q (Q1–7, Q10, Q11) and the Takakura-Tanaka classification, osteophyte score, mean HU ratio of all regions, synovial thickness score, and total BME were examined (Table 5). The HU ratio showed moderate correlations with the points for all questions. The synovial thickness score differed from the Takakura-Tanaka classification stage, osteophyte score, and total BME in that the correlation coefficient was greater for pain at rest, such as questions 2 and 6, than for pain while walking, such as questions 10 and 11.

Considering that OA severity can be a confounding factor for HU ratios, we also examined the correlation between pain and pain-related score and HU ratios at each stage of the Takakura-Tanaka classification (Table 6). In stage 1, the HU ratio in the medial gutter showed a significant correlation with the pain and pain-related score. In stage 2, this correlation extended to the medial part of the tibiotalar joint. In stage 3 A and above, a significant correlation was observed in the central or lateral parts of the tibiotalar joint.

All measurements demonstrated high reliability. Radiographic angular measurements showed excellent reliability (ICC (1,1) = 0.992, 95% confidence interval (CI) 0.982–0.990; ICC (2,1) = 0.988, 95% CI 0.970–0.995). The total osteophyte score also showed excellent reliability (ICC (1,1) = 0.962, 95% CI 0.917–0.980; ICC (2,1) = 0.949, 95% CI 0.889–0.970). The HU ratio demonstrated good to excellent reliability (ICC (1,1) = 0.915, 95% CI 0.898–0.920; ICC (2,1) = 0.840, 95% CI 0.811–0.860), as did synovial thickness score (ICC (1,1) = 0.923, 95% CI 0.835–0.960; ICC (2,1) = 0.886, 95% CI 0.757–0.940) and total BME (ICC (1,1) = 0.933, 95% CI 0.855–0.970; ICC (2,1) = 0.888, 95% CI 0.763–0.940).

**Table 1:** Comparison of demographics, physical findings, and SAFE-Q subscale scores between the severe and mild pain groups (mean ± standard deviation (range)). *CLAI* chronic lateral ankle instability

	All patients (*n*=75)	Severe pain group (*n*=37)	Mild pain group (*n*=38)	*P* value
Sex, male/female, number	32/43	14/23	18/20	0.817
Side, right/left, number	45/30	20/17	25/13	0.351
Etiology, CLAI-associated/primary, number	62/13	28/9	34/4	0.137
Age, y	57.3 ± 15.1 (20–88)	**62.2 ± 13.3 (24–83)**	**52.6 ± 15.2 (20–88)**	**< 0.01**
Body mass index, kg/m²	24.6 ± 4.1 (16.2–39.5.2.5)	**25.4 ± 4.0 (16.2–34.9.2.9)**	**23.7 ± 4.0 (17.4–39.5.4.5)**	**< 0.05**
Dorsiflexion range of motion, degrees	19.2 ± 8.1 (0–30)	17.2 ± 9.1 (0–30)	21.1 ± 6.5 (0–30)	0.129
Plantar flexion range of motion, degrees	41.9 ± 7.6 (15–50)	39.9 ± 9.5 (15–50)	43.9 ± 4.4 (25–50)	0.129
Self-Administered Foot Evaluation Questionnaire				
Pain and pain-related score, points	46.4 ± 19.3 (5.6–82.2.6.2)	**30.1 ± 11.0 (5.6–45.6.6.6)**	**62.3 ± 10.3 (47.2–82.2.2.2)**	**< 0.01**
Physical functioning and daily living score, points	58.4 ± 22.2 (9.1–97.7.1.7)	**44.9 ± 18.4 (9.1–79.6.1.6)**	**71.5 ± 17.2 (31.8–97.7.8.7)**	**< 0.01**
Social functioning score, points	54.3 ± 28.0 (0.0–100.0.0.0)	**39.4 ± 24.8 (0.0–95.8.0.8)**	**69.1 ± 22.5 (12.5–100.0.5.0)**	**< 0.01**
Shoe-related score, points	62.6 ± 28.7 (0.0–100.0.0.0)	**49.1 ± 29.0 (0.0–100.0.0.0)**	**75.7 ± 21.4 (16.7–100.0.7.0)**	**< 0.01**
General health and well-being score, points	50.1 ± 28.1 (0.0–100.0.0.0)	**36.4 ± 23.4 (5.0–100.0.0.0)**	**63.6 ± 25.8 (0.0–100.0.0.0)**	**< 0.01**
Visual analog scale, points	6.1 ± 2.5 (1–10)	**8.0 ± 1.3 (5–10)**	**4.2 ± 1.9 (1–9)**	**< 0.01**

The bold values mean that there are significant differences

**Table 2:** Comparison of radiographic and imaging findings between the severe and mild pain groups (mean ± standard deviation (range))

	All patients (*n*=75)	Severe pain group (*n*=37)	Mild pain group (*n*=38)	*P* value
Takakura-Tanaka classification grade (1/2/3A/3B/4)	18/13/19/16/9	**3/4/10/12/8**	**15/9/9/4/1**	**< 0.01**
Tibial anterior surface angle, degrees	85.3 ± 3.1 (74.0–90.4.0.4)	84.8 ± 3.4 (74.0–90.4.0.4)	85.9 ± 2.7 (80.7–90.0.7.0)	0.159
Tibial lateral surface angle, degrees	80.6 ± 3.5 (68.7–88.9.7.9)	80.2 ± 3.2 (71.3–85.9.3.9)	81.0 ± 3.6 (68.7–88.9.7.9)	0.209
Talar tilt ankle, degrees	4.9 ± 5.5 (0.0–20.4.0.4)	**6.5 ± 5.9 (0.0–20.4.0.4)**	**3.5 ± 4.6 (0.0–16.6.0.6)**	**< 0.05**
Tibiomedial malleolar angle, degrees	38.2 ± 11.7 (18.4–64.6.4.6)	**41.9 ± 12.7 (18.4–64.6.4.6)**	**34.7 ± 9.4 (20.8–54.9.8.9)**	**< 0.05**
Total osteophyte score, points	16.1 ± 6.9 (2–30)	**19.3 ± 6.4 (4–30)**	**13.0 ± 5.8 (2–25)**	**< 0.01**
Hounsfield unit ratio				
Medial gutter of the tibia	4.3 ± 2.4 (1.6–18.0.6.0)	**5.5 ± 2.8 (2.5–18.0.5.0)**	**3.2 ± 1.1 (1.6–6.5.6.5)**	**< 0.01**
Medial gutter of the talus	4.6 ± 2.5 (1.9–19.1.9.1)	**5.7 ± 3.0 (2.5–19.1.5.1)**	**3.4 ± 1.1 (1.9–7.0.9.0)**	**< 0.01**
Medial part of the tibial plafond	4.2 ± 2.3 (1.6–14.9.6.9)	**5.4 ± 2.6 (2.3–14.9.3.9)**	**3.0 ± 0.9 (1.6–6.1.6.1)**	**< 0.01**
Central part of the tibial plafond	3.6 ± 2.3 (1.4–18.3.4.3)	**4.7 ± 2.8 (1.7–18.3.7.3)**	**2.5 ± 0.9 (1.4–5.4.4.4)**	**< 0.01**
Lateral part of the tibial plafond	3.1 ± 1.6 (1.4–10.3.4.3)	**3.8 ± 1.9 (1.4–10.3.4.3)**	**2.3 ± 0.6 (1.5–4.4.5.4)**	**< 0.01**
Medial part of the talar dome	4.4 ± 2.4 (1.7–14.6.7.6)	**5.7 ± 2.8 (2.1–14.6.1.6)**	**3.2 ± 1.0 (1.7–6.1.7.1)**	**< 0.01**
Central part of the talar dome	3.6 ± 1.8 (1.6–12.0.6.0)	**4.5 ± 2.1 (1.9–12.0.9.0)**	**2.7 ± 0.8 (1.6–5.4.6.4)**	**< 0.01**
Lateral part of the talar dome	3.2 ± 1.5 (1.7–9.1.7.1)	**4.0 ± 1.7 (2.0–9.1.0.1)**	**2.5 ± 0.6 (1.7–4.2.7.2)**	**< 0.01**
Lateral gutter of the talus	2.5 ± 1.2 (1.4–9.3.4.3)	**3.0 ± 1.5 (1.5–9.3.5.3)**	**2.1 ± 0.5 (1.5–3.6.5.6)**	**< 0.01**
Lateral gutter of the fibula	2.2 ± 1.0 (0.7–7.6.7.6)	2.4 ± 1.3 (0.8–7.6.8.6)	2.0 ± 0.6 (0.7–13.9.7.9)	0.384
Mean of all regions	3.5 ± 1.6 (1.7–10.9.7.9)	**4.3 ± 1.8 (2.0–10.9.0.9)**	**2.7 ± 0.7 (1.7–4.7.7.7)**	**< 0.01**
Synovial thickness score, points	5.4 ± 2.0 (2–8)	**6.0 ± 1.9 (2–8)**	**4.8 ± 1.9 (2–8)**	**< 0.01**
Total bone marrow edema, mm²	256.1 ± 374.6 (0.0–2340.3.0.3)	**361.5 ± 422.0 (0.0–2340.3.0.3)**	**153.4 ± 286.8 (0.0–1501.4.0.4)**	**< 0.01**

The bold values mean that there are significant differences

**Table 3:** Correlations between pain and pain-related score and age, physical findings, and image findings

	*r*	*P* value
Age	**− 0.331**	**< 0.01**
Body mass index	− 0.205	0.081
Dorsiflexion range of motion	0.225	0.055
Plantar flexion range of motion	**0.349**	**< 0.01**
Takakura-Tanaka classification stage	**− 0.529**	**< 0.01**
Tibial anterior surface angle	0.162	0.166
Tibial lateral surface angle	0.102	0.383
Talar tilt ankle	**− 0.241**	**< 0.05**
Tibiomedial malleolar angle	**− 0.362**	**< 0.01**
Osteophyte score	**− 0.460**	**< 0.01**
HU ratio medial part of the tibial plafond	**− 0.734**	**< 0.01**
HU ratio central part of the tibial plafond	**− 0.689**	**< 0.01**
HU ratio lateral part of the tibial plafond	**− 0.681**	**< 0.01**
HU ratio medial part of the talar dome	**− 0.721**	**< 0.01**
HU ratio central part of the talar dome	**− 0.671**	**< 0.01**
HU ratio lateral part of the talar dome	**− 0.656**	**< 0.01**
HU ratio medial gutter of the tibia	**− 0.676**	**< 0.01**
HU ratio medial gutter of the talus	**− 0.656**	**< 0.01**
HU ratio lateral gutter of the talus	**− 0.535**	**< 0.01**
HU ratio lateral gutter of the fibula	− 0.212	0.067
HU ratio mean of the all region	**− 0.729**	**< 0.01**
Synovial thickness score	**− 0.387**	**< 0.01**
Total bone marrow edema	**− 0.475**	**< 0.01**

The bold values mean that there are significant differences

**Table 4:** Multiple regression analysis for pain and pain-related score

	Variance Inflation Factor	Regression coefficient	T value	*P* value
Age	1.767	0.044	0.315	0.754
Body mass index	1.156	− 0.548	−1.298	0.199
Talar tilt angle	1.662	0.095	0.254	0.8
Takakura-Tanaka classification	**2.961**	**−6.707**	**−2.344**	**< 0.05**
Osteophyte score	3.397	0.335	0.788	0.434
Hounsfield unit ratio	**1.475**	**−5.427**	**−4.449**	**< 0.01**
Synovial thickness score	**1.201**	**−1.872**	**−2.117**	**< 0.05**
Total bone marrow edema	1.199	− 0.003	− 0.561	0.577

The bold values mean that there are significant differences

**Table 5:** Correlations between each question of the pain and pain-related score and each parameter

Questionnaire	Points	Takakura-Tanaka classification		Osteophyte score		Mean HU ratio of all regions		Synovial thickness score		Total BME	
	mean ± standard deviation (range)	*r*	*P* value	*r*	*P* value	*r*	*P* value	*r*	*P* value	*r*	*P* value
Q1. Have you noticed any pain in your foot (feet) during the past week?	2.9 ± 1.1 (0–4)	**0.321**	**< 0.01**	0.224	0.0531	**0.494**	**< 0.01**	0.220	0.058	**0.263**	**< 0.05**
Q2. Have you had difficulty in sleeping due to foot pain in the past week?	1.0 ± 1.1 (0–4)	**0.254**	**< 0.05**	0.212	0.067	**0.481**	**< 0.01**	**0.248**	**< 0.05**	0.146	0.211
Q3. How intense was the most severe pain you experienced in your feet in the past week? (VAS)	6.1 ± 2.5 (1–10)	**0.523**	**< 0.01**	**0.481**	**< 0.01**	**0.677**	**< 0.01**	**0.361**	**< 0.01**	**0.524**	**< 0.01**
Q4. How intense was the foot pain you experienced while walking on flat ground in the past week?	2.1 ± 1.1 (1–4)	**0.505**	**< 0.01**	**0.442**	**< 0.01**	**0.531**	**< 0.01**	**0.466**	**< 0.01**	**0.422**	**< 0.01**
Q5. Have you had foot pain in the past week?	2.9 ± 1.3 (0–4)	**0.392**	**< 0.01**	**0.287**	**< 0.05**	**0.485**	**< 0.01**	**0.281**	**< 0.05**	**0.372**	**< 0.01**
Q6. How intense was the foot pain you experienced when you woke up in the morning in the past week?	1.7 ± 1.3 (0–4)	0.201	0.083	0.188	0.107	**0.437**	**< 0.01**	**0.277**	**< 0.05**	**0.285**	**< 0.05**
Q7. How intense was the foot pain you experienced at the end of each day in the morning in the past week?	2.4 ± 1.2 (0–4)	**0.470**	**< 0.01**	**0.438**	**< 0.01**	**0.660**	**< 0.01**	**0.270**	**< 0.05**	**0.372**	**< 0.01**
Q10. How intense was the foot pain you experienced while walking barefoot in the past week?	2.1 ± 1.0 (0–4)	**0.475**	**< 0.01**	**0.452**	**< 0.01**	**0.622**	**< 0.01**	**0.232**	**< 0.05**	**0.485**	**< 0.01**
Q11. How intense was the foot pain you experienced while walking in shoes in the past week?	2.0 ± 0.9 (1–4)	**0.575**	**< 0.01**	**0.471**	**< 0.01**	**0.567**	**< 0.01**	0.225	0.052	**0.416**	**< 0.01**

The bold values mean that there are significant differences

**Table 6:** Correlations between the pain and pain-related score and Hounsfield unit ratio in each stage

Region	stage 1		stage 2		stage 3 A		stage 3B		stage 4	
	*r*	*P* value	*r*	*P* value	*r*	*P* value	*r*	*P* value	*r*	*P* value
Medial gutter										
Tibia	**− 0.505**	**< 0.05**	**− 0.775**	**< 0.01**	**− 0.551**	**< 0.05**	**− 0.501**	**< 0.05**	**− 0.767**	**< 0.05**
Talus	**− 0.490**	**< 0.05**	**− 0.621**	**< 0.05**	**− 0.618**	**< 0.01**	**− 0.599**	**< 0.05**	− 0.683	0.050
Tibial plafond										
Medial part	− 0.459	0.0552	**− 0.632**	**< 0.05**	**− 0.781**	**< 0.01**	**− 0.542**	**< 0.05**	**− 0.783**	**< 0.05**
Central part	− 0.209	0.4041	− 0.522	0.0707	**− 0.706**	**< 0.01**	**− 0.566**	**< 0.05**	− 0.650	0.0666
Lateral part	− 0.135	0.5927	− 0.555	0.0525	**− 0.708**	**< 0.01**	**− 0.677**	**< 0.01**	− 0.650	0.0666
Talar dome										
Medial part	− 0.226	0.3672	**− 0.604**	**< 0.05**	**− 0.696**	**< 0.01**	**− 0.607**	**< 0.05**	**− 0.833**	**< 0.01**
Central part	− 0.263	0.2914	− 0.489	0.093	**− 0.584**	**< 0.01**	**− 0.591**	**< 0.05**	**− 0.733**	**< 0.05**
Lateral part	− 0.199	0.4282	**− 0.571**	**< 0.05**	**− 0.591**	**< 0.01**	**− 0.608**	**< 0.05**	− 0.667	0.0589
Lateral gutter										
Talus	− 0.104	0.6806	− 0.522	0.0707	− 0.415	0.077	− 0.401	0.1246	***− 0.867***	***< 0.01***
Fibula	− 0.057	0.823	− 0.440	0.135	− 0.379	0.1093	− 0.016	0.954	− 0.400	0.2912

The bold values mean that there are significant differences

## Discussion

### Correlation of imaging findings with pain

This study showed that subchondral bone sclerosis assessed by CT using HU values, synovitis evaluated on MRI, and radiographic OA severity are significantly correlated with pain in patients with varus ankle OA. Notably, subchondral bone sclerosis showed the strongest correlation with pain regardless of OA severity, even in areas where the joint space appeared preserved on radiographs.

The association of synovitis with OA pain is well-accepted and widely reported in the literature [25]. The production of cytokines and inflammatory mediators sensitizes sensory nerves and thickened synovium or effusion mechanically stimulate nociceptors [8, 26]. Chronic inflammation in the joint also contributes to accelerated cartilage damage, which is associated with joint pain and the progression of OA. Regarding bone lesions, the association between BME and OA pain is well known [8, 27], and this study also demonstrated a moderate correlation. However, HU ratios demonstrated a stronger correlation with pain than BME, indicating that subchondral bone sclerosis is more closely linked to ankle OA pain.

We speculate that the association between the subchondral sclerosis and OA pain is mediated by three mechanisms. First, long-term mechanical joint stress due to weight loading and ankle instability may promote subchondral bone sclerosis, relating to pain. Previous reports indicate that subchondral bone sclerosis and associated pain occur even in the early stages [21, 23]. The ankle joint is exposed to more weight loading than the knee and hip [28–30], and varus ankle OA is closely associated with lateral ankle instability, leading to high stress. Second, subchondral bone sclerosis directly affects cartilage degeneration and the progression of OA [31–33]. Cartilage damage or loss increases the mechanical load on the underlying bone, which can be related to joint pain [34].

Third, nervous innervation and vascularization of cartilage originating from the subchondral bone may contribute to OA pain, with neuropeptides such as substance P and calcitonin gene-related peptide playing key roles [7]. Subchondral bone sclerosis and associated cartilage damage may be related to pain via stimulation of sensory nerves innervating the cartilage and excessive neuropeptide secretion.

**Fig. 4:**
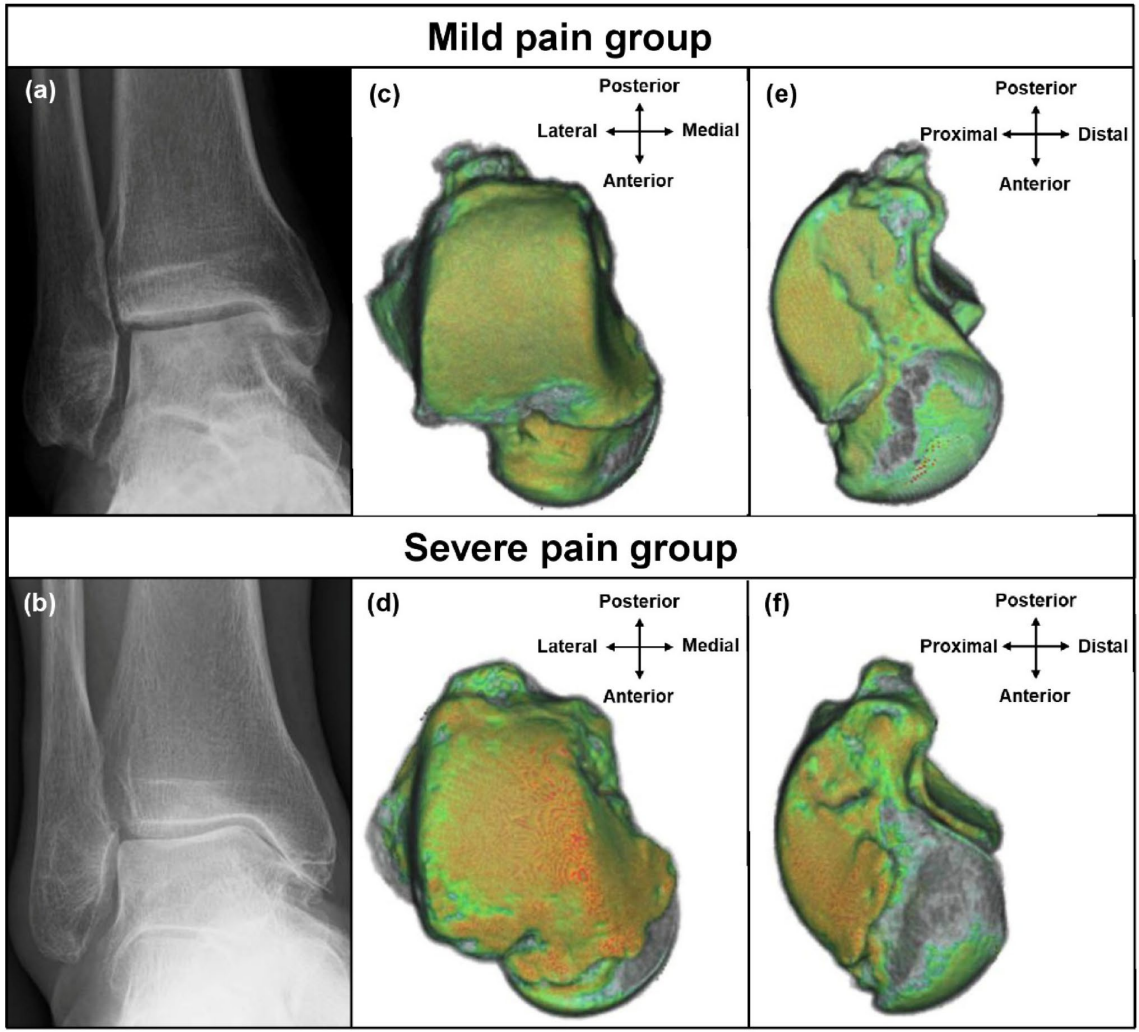
Representative images of the mild and severe pain groups. **a**, **b** Weight-bearing plain radiograph of stage 3 A ankle osteoarthritis. Colorization of Hounsfield unit (HU) values on three-dimensional computed tomography images of the talar dome (**c**, **d**) and medial gutter of the talus (**e**,** f**). HU values: red >orange >green >blue >colorless

Furthermore, the HU ratios were significantly correlated with pain not only in areas with joint spaces narrowing, but also in areas where the joint space was preserved: the medial part of the tibiotalar joint in stage 2, the tibiotalar joint in stage 3 A, and the central and lateral parts of the tibiotalar joint in stage 3B. A previous report showed similar findings: the HU ratios of the medial gutter are correlated with pain in patients with CLAI, including Takakura-Tanaka stage 1 ankle OA [9]. In varus ankle OA with severe pain, subchondral bone sclerosis may have already progressed even when the joint space appears preserved on plain radiographs (Fig. 4). This may suggest the possibility of more advanced underlying cartilage degeneration than predicted by radiographic findings.

### Clinical implications

The mechanisms underlying pain in ankle OA are multifactorial and cannot be explained by radiographic severity alone. Severe pain with relatively preserved joint space can reflect synovitis, mechanical stress, subchondral bone sclerosis, or potential cartilage degeneration. CT and MRI can provide valuable information, but even when such imaging is not feasible, severe pain itself should alert clinicians to possible progression beyond radiographic findings. Such patients may require early intervention not only for pain relief but also to prevent functional disability resulting from further progression of OA. For example, for stage 1 or 2 OA with severe pain, lateral ligament repair, synovectomy, or supramalleolar osteotomy (SMO) may be actively recommended. In contrast, for patients with stage 3 A or 3B OA and severe pain, the indication for SMO should be carefully determined, as a lateral shift of the mechanical axis may disrupt the potentially degenerated cartilage in the central and lateral parts. We consider SMO to be an appropriate indication for stage 3 A OA. However, careful preoperative MRI and intraoperative arthroscopic evaluation of cartilage is advisable, with conversion to other procedures, such as arthrodesis or total ankle arthroplasty, considered if advanced degeneration is detected. Based on our findings, in stage 3B OA with severe pain, cartilage degeneration of the central and lateral parts is expected to be advanced, and SMO may best be avoided.

### Limitations

This study has several limitations. First, it was retrospective, single-center, and incvolved a relatively small cohort, which may limit generalizability. Second, as it included only patients who underwent surgery, those with mild symptoms not requiring surgery were excluded. Therefore, the findings may not be generalizable to earlier OA stages. However, performing examinations with high costs and radiation exposure in such patients was difficult. Third, we excluded valgus OA and post-traumatic OA. Although there are many cases of valgus and post-traumatic OA, this study intentionally included only CLAI with OA findings and varus ankle OA to unify the pathology. Fourth, pain caused by extra-articular pathologies, such as nerve sensitization and psychological influences, cannot be ruled out. The causes of OA pain are complex and varied, making it difficult to evaluate and exclude. Fifth, the use of analgesics and other pain-modulating treatments remains unclear. As patients with OA experience a long clinical course from pain onset to surgery, information about the entire treatment course was difficult. Sixth, although we interpreted increased HU ratios as reflecting subchondral sclerosis and potential cartilage degeneration, our study did not directly correlate them with intraoperative or histological cartilage findings. Finally, two MRI systems (1.5T and 3.0T) were used, which may have introduced variability. The 1.5T MRI system had thin slice (0.8 mm) that likely mitigated significant differences in image quality. Further research is needed in the future to address these limitations.

## Conclusion

Ankle pain in patients with varus ankle OA is significantly correlated with subchondral bone sclerosis of the tibia and talus, radiographic OA severity, and synovitis. Notably, subchondral bone sclerosis correlated most strongly and at every OA stage. Severe pain can indicate high mechanical stress and subchondral bone sclerosis associated with cartilage degeneration. Varus ankle OA with severe pain should be managed early and appropriately, regardless of radiographic severity.

## Data Availability

No datasets were generated or analysed during the current study.
